# Resolution of shawl sign in dermatomyositis using pulsed dye laser

**DOI:** 10.1016/j.jdcr.2021.07.032

**Published:** 2021-08-19

**Authors:** Marie-Eline Pauline Henriette Debeuf, Marloes van Onna, Valerie Lydie Roland Marc Verstraeten

**Affiliations:** a Department of Dermatology, Maastricht University Medical Centre, Maastricht, Limburg, the Netherlands; b Division of Rheumatology, Department of Internal Medicine, Maastricht University Medical Centre, Maastricht, Limburg, the Netherlands; c School for Public Health and Primary Care (CAPHRI), Maastricht University, Maastricht, Limburg, the Netherlands; d GROW School for Oncology and Developmental Biology, Maastricht University, Maastricht, Limburg, the Netherlands

**Keywords:** dermatomyositis, poikiloderma, pulsed dye laser, shawl sign, PDL, pulsed dye laser

## Introduction

Dermatomyositis is an inflammatory myopathy that affects children and adults. It can present without muscle involvement in the case of amyopathic dermatomyositis. Whereas the juvenile variant is often associated with calcinosis cutis, the adult variant is typically associated with malignancies.^1^ Approximately 94% of the patients with dermatomyositis develop cutaneous manifestations.^2^^,^^3^ Typical skin findings include the Gottron sign, which refers to a symmetrical heliotropic livid-erythematous macule on the face often including the eyelids, Gottron papules on the elbows and metacarpal or interphalangeal joints of the hands, nailfold changes characterized by ragged cuticulae and enlarged telangiectasias in the nail bed, and poikilodermatous skin changes in the neck and on the chest (shawl sign). Histopathology reveals epidermal atrophy, vacuolar changes of the basal epidermal cell layer, hyperkeratosis, mucin deposition, basement membrane thickening, dermal edema, pigment incontinence, and a perivascular infiltrate.^4^ Treatment includes topical and/or systemic corticosteroids. Hereafter, steroid-sparing therapy is initiated with methotrexate, hydroxychloroquine, azathioprine, tacrolimus, cyclosporine A, mycophenolate mofetil, intravenous immunoglobulin, or rituximab.^5^ Despite therapy, symptoms such as pain and burning sensation associated with skin lesions can be therapy-resistant and affect the quality of daily life. Laser therapy is an emerging treatment in inflammatory skin diseases for which only very few reports exist on dermatomyositis. Here, we report the resolution of the shawl sign with 2 sessions of pulsed dye laser (PDL).

## Case report

**Fig 1:**
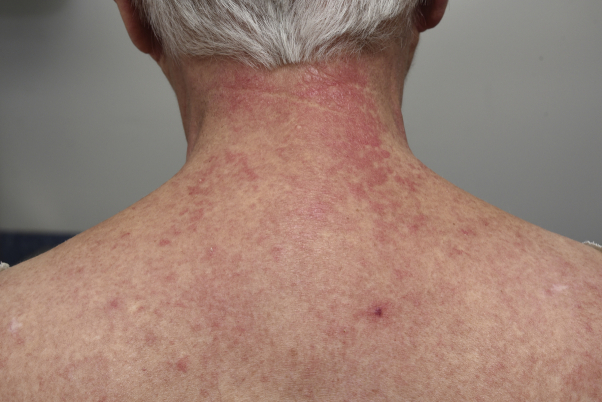
Livid-erythematous poikiloderma (shawl sign) in the neck before treatment.

**Fig 2:**
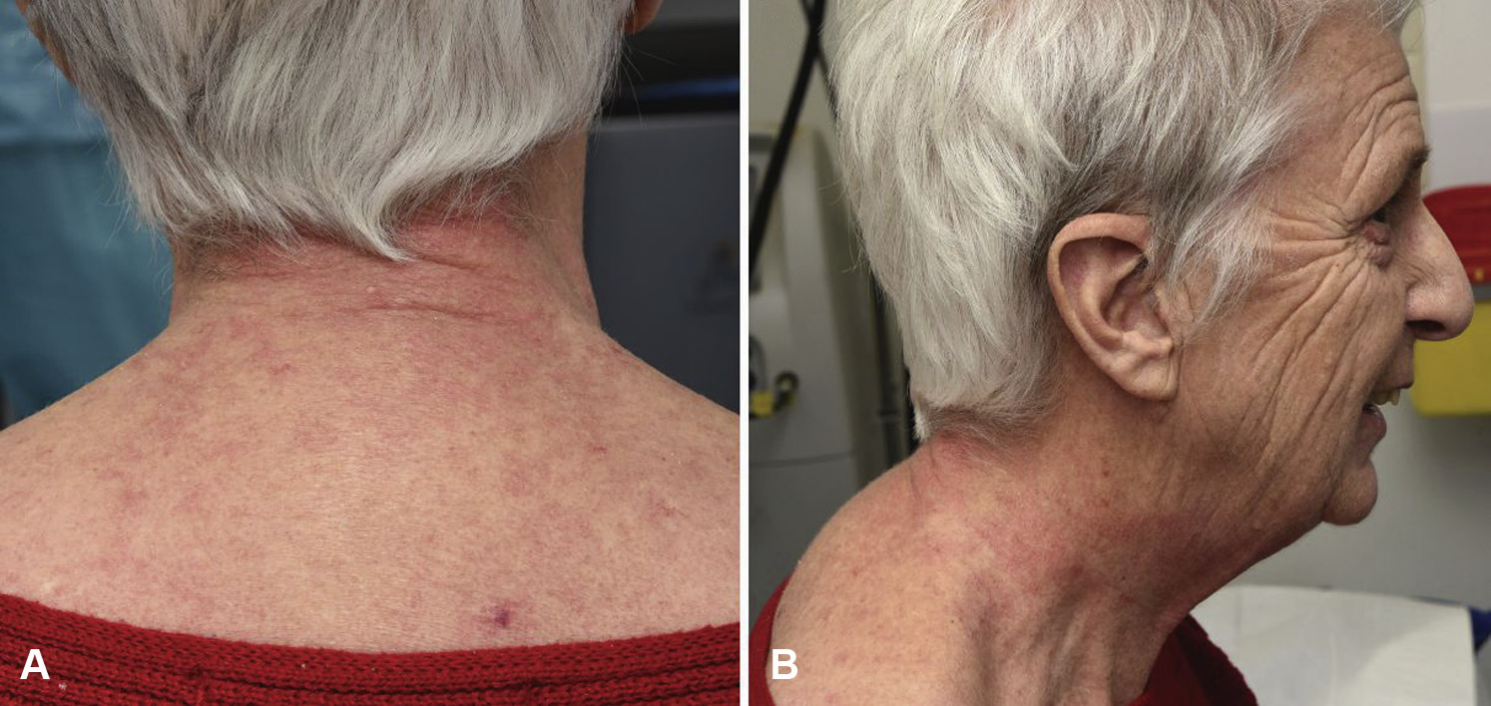
**A** and **B**, Great improvement of the shawl sign after a single treatment using pulsed dye laser.

**Fig 3:**
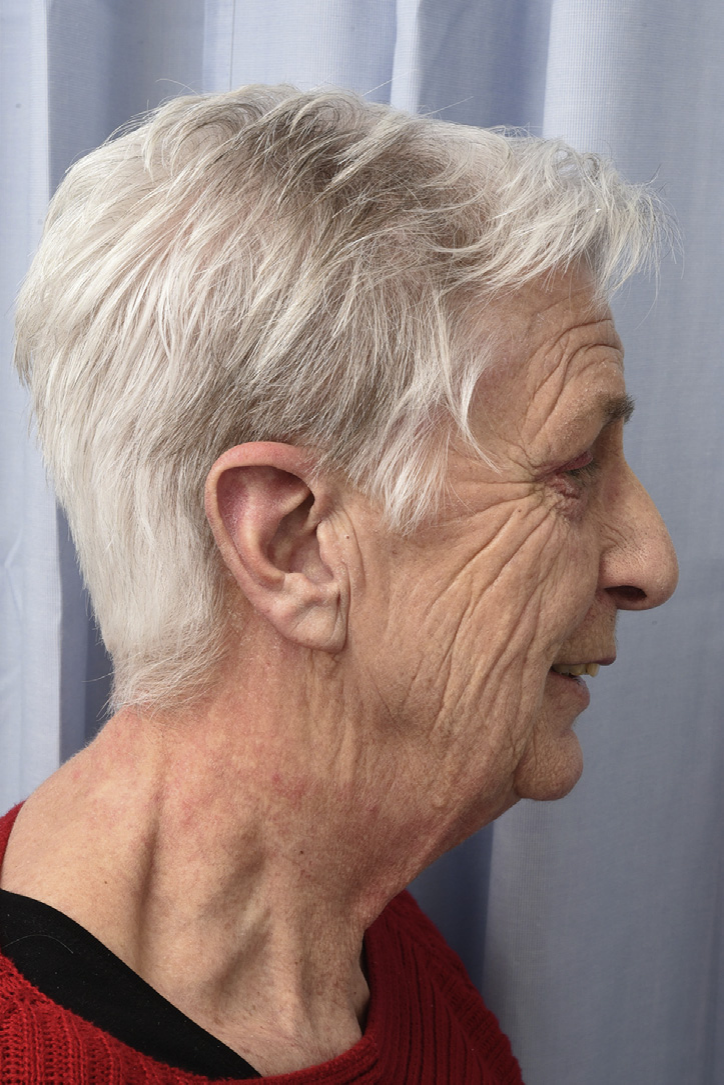
Remission of the shawl sign upon 2 treatments with pulsed dye laser.

**Fig 4:**
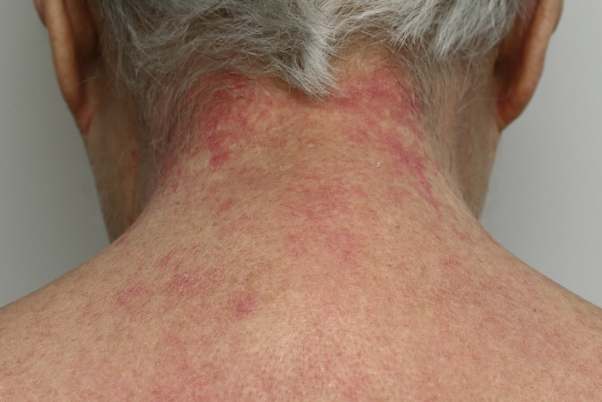
One year after pulsed dye laser therapy, part of the shawl sign reoccurred.

A 72-year-old patient with a 6-year history of amyopathic dermatomyositis presented with livid-red poikiloderma in the neck (shawl sign), Gottron sign on the face, and Gottron papules on the hands. The scalp showed some discrete erythematosquamous plaques as well as many irregular telangiectasias and partial alopecia parietally. Importantly, the patient experienced a burning sensation in the neck. The skin findings, other than the shawl sign, responded reasonably well and stabilized with 20-mg methotrexate per week. Additionally, betamethasone-calcipotriol ointment was prescribed for the scalp and betamethasone dipropionate ointment for the neck and upper portion of arms. Unfortunately, the poikiloderma in the neck caused a burning sensation that remained therapy-resistant. Subsequently, we performed PDL therapy of the livid-erythematous and poikilodermatous patches on the neck (Fig 1). Treatment resulted in a decrease in erythema (Fig 2, *A* and *B*), and the burning sensation improved dramatically with the first PDL treatment. Seven weeks later, we performed the second PDL treatment of the remaining poikiloderma in the neck, which resulted in complete resolution (Fig 3). Aside from a temporary burning sensation during laser therapy and some mild discomfort in the first 4 days following PDL therapy, the patient experienced no other side effects. PDL therapy (595-nm wavelength) was performed using a 7-mm spot size, 8 J and 1.5 ms, 1 pass. By use of a cryogen-based dynamic cooling device, the epidermis was cooled at each pulse. The clinical end point was a persistent gray macule following the laser pulse, eventually resulting in a purpuric macule. Most of the purpura resolved 1 week after laser treatment, the remaining purpura in the week hereafter. Following laser therapy, the patient was instructed to apply cool packs for 10 minutes every hour for the first 2 days. After these 2 PDL laser sessions, the patient remained in remission for more than a year. Thereafter, reactivation of the shawl sign partially occurred (Fig 4). There were no such side effects as hyperpigmentation, hypopigmentation, or scarring.

## Discussion

This case report illustrates successful treatment of poikilodermatous erythema in the neck (shawl sign) using PDL in a patient with longstanding dermatomyositis. Regardless of topical corticosteroids and systemic immune suppressive therapy, poikiloderma remains therapy-resistant and causes a burden to the patient's quality of life. Strikingly, a single PDL session can provide an extensive improvement in both subjective and objective symptoms, improving not only the livid-erythematous skin lesions but also the associated pain and burning sensation. In contrast to reports on laser therapy as a valuable additive to the treatment of lupus erythematosus, only 6 case reports exist on laser therapy in dermatomyositis.^6^, ^7^, ^8^, ^9^, ^10^, ^11^ Most of these reports consider the effect of the carbon dioxide laser and picosecond laser in the treatment of calcinosis cutis. The PDL, argon laser, and intense pulse light system have been reported in the treatment of telangiectasias, chronic facial erythema, poikiloderma, and Gottron papules in dermatomyositis. In our patient, therapy-resistant livid-erythematous poikiloderma was treated with PDL. Poikiloderma consists of hyperpigmentation, atrophy, and telangiectasias. These blood vessels are well-targeted by PDL laser therapy since the 595-nm light is greatly absorbed by hemoglobin. Reported adverse reactions when using the PDL include pain during treatment, posttreatment hyperpigmentation, hypopigmentation, and scarring. By use of cooling systems after treatment, the risk for these adverse reactions can be minimized.^12^ In our patient, the purpuric posttreatment macules resolved without long-term dyspigmentation and scarring. Importantly, the patient not only experienced cosmetic improvement but also relief of the burning sensation that was associated with the poikiloderma. The decrease in itch and prickling could result from a reduction in transforming growth factor β and CD4^+^ T cells after PDL. Moreover, transforming growth factor β induces the expression of interleukin 31, a cytokine that stimulates itch, and interleukin 31 is mainly expressed by activated CD4^+^ T cells.^13^, ^14^, ^15^, ^16^ Whereas current guidelines^17^^,^^18^ would advise increasing the topical or systemic immune modulatory drugs in efforts to eradicate such persistent skin lesions with a considerable burden on the quality of life, laser therapy should be taken into consideration as an easy way to treat these and avoid the need for additional immunosuppressive drugs.

## Conflicts of interest

None disclosed.
